# Ethylene Receptors Function as Components of High-Molecular-Mass Protein Complexes in *Arabidopsis*

**DOI:** 10.1371/journal.pone.0008640

**Published:** 2010-01-08

**Authors:** Yi-Feng Chen, Zhiyong Gao, Robert J. Kerris, Wuyi Wang, Brad M. Binder, G. Eric Schaller

**Affiliations:** 1 Department of Biological Sciences, Dartmouth College, Hanover, New Hampshire, United States of America; 2 Department of Botany, University of Wisconsin, Madison, Wisconsin, United States of America; University of Heidelberg, Germany

## Abstract

**Background:**

The gaseous plant hormone ethylene is perceived in *Arabidopsis thaliana* by a five-member receptor family composed of ETR1, ERS1, ETR2, ERS2, and EIN4.

**Methodology/Principal Findings:**

Gel-filtration analysis of ethylene receptors solubilized from *Arabidopsis* membranes demonstrates that the receptors exist as components of high-molecular-mass protein complexes. The ERS1 protein complex exhibits an ethylene-induced change in size consistent with ligand-mediated nucleation of protein-protein interactions. Deletion analysis supports the participation of multiple domains from ETR1 in formation of the protein complex, and also demonstrates that targeting to and retention of ETR1 at the endoplasmic reticulum only requires the first 147 amino acids of the receptor. A role for disulfide bonds in stabilizing the ETR1 protein complex was demonstrated by use of reducing agents and mutation of Cys4 and Cys6 of ETR1. Expression and analysis of ETR1 in a transgenic yeast system demonstrates the importance of Cys4 and Cys6 of ETR1 in stabilizing the receptor for ethylene binding.

**Conclusions/Significance:**

These data support the participation of ethylene receptors in obligate as well as ligand-dependent non-obligate protein interactions. These data also suggest that different protein complexes may allow for tailoring of the ethylene signal to specific cellular environments and responses.

## Introduction

The gaseous plant hormone ethylene (C_2_H_4_) regulates a broad spectrum of developmental and physiological processes including germination, growth, senescence, ripening, and responses to biotic and abiotic stress [1], [2]. In Arabidopsis, ethylene is perceived by a receptor family composed of ETR1, ERS1, ETR2, ERS2, and EIN4 [3], [4], [5]. The ethylene receptors have a similar overall modular structure, each containing three conserved transmembrane domains near the N-terminus, followed by a GAF domain of unknown function, and then signal output motifs in the C-terminal half. Although similar, the ethylene receptors can be divided into two subfamilies based on phylogenetic analysis and some shared structural features, subfamily 1 being composed of ETR1 and ERS1, subfamily 2 being composed of ETR2, ERS2, and EIN4 [3], [5], [6].

The N-terminal region of the receptors is involved in membrane localization, ethylene binding, and dimerization. One purpose of the transmembrane domains is localization of the receptors to the endoplasmic reticulum, an unusual location for a hormone receptor but one compatible with the ready diffusion of ethylene in aqueous and lipid environments [7], [8], [9]. Genetic and biochemical evidence indicate that the transmembrane domains also contain the ethylene-binding site, with binding requiring the presence of a copper cofactor [4], [10], [11], [12]. The basic functional unit for ethylene perception is apparently a dimer, based on the finding that there is one copper ion, and thus the ability to bind one molecule of ethylene, per receptor dimer [11]. Consistent with a dimer being the functional unit is the finding that two receptor monomers are maintained as a disulfide-linked dimer, two conserved Cys residues near the N-terminus being implicated in forming the covalent linkage [13], [14].

In the C-terminal half of each receptor are domains with similarity to His kinases and in some cases the receiver domains of response regulators. His kinases and receiver domains are signaling elements originally identified in bacterial two-component phosphorelays and are now known to be present in plants, fungi, and slime molds [15]. His kinase activity has been confirmed in vitro for the subfamily-1 receptors ETR1 and ERS1, which contain all the residues considered essential for enzymatic activity [16], [17]. His kinase activity has not been detected in the subfamily-2 receptors ETR2, ERS2, and EIN4; these lack residues considered essential for His kinase activity and instead are now thought to act as Ser/Thr kinases [17]. The subfamily-1 receptors of Arabidopsis play the predominant role in ethylene signaling [18], [19], but the degree to which His kinase activity contributes to ethylene signal transduction is not resolved, although it has been implicated in modulating both the establishment of and the recovery from the ethylene response [18], [20], [21].

The ethylene receptors are present at very low abundance, rendering purification to homogeneity impractical for functional characterization and for the identification of interacting components. As a result, much of the functional characterization has relied upon heterologous expression systems, such as the use of transgenic yeast or bacteria to characterize ethylene binding and kinase activity [22], [23]. In addition, because other elements of the signal transduction pathway have been identified by genetic analysis, these have been characterized for their ability to localize to the endoplasmic reticulum and to interact with the receptors. Among the downstream pathway components implicated in forming physical interactions with the receptors are CTR1, a Raf-like protein kinase [24], [25], [26], [27], and EIN2, a transmembrane protein related to a class of metal transporters [28]. Furthermore, the ethylene receptors themselves have been demonstrated to interact with each other to form higher-order receptor complexes [9], [29]. These studies support the concept that multiprotein complexes are the functional units for signal transduction by the ethylene receptors. Here we describe complementary evidence obtained from gel filtration chromatography of solubilized receptors, which indicates that the receptors function as components within high-molecular-mass protein complexes, that differences exist among the protein complexes formed by different members of the receptor family, that disulfide linkages play a role in stabilizing the receptor complexes, and that novel components within the complexes still remain undiscovered.

## Results

### ETR1 Is Isolated as Part of a High-Molecular-Mass Protein Complex from *Arabidopsis*

**Figure 1:**
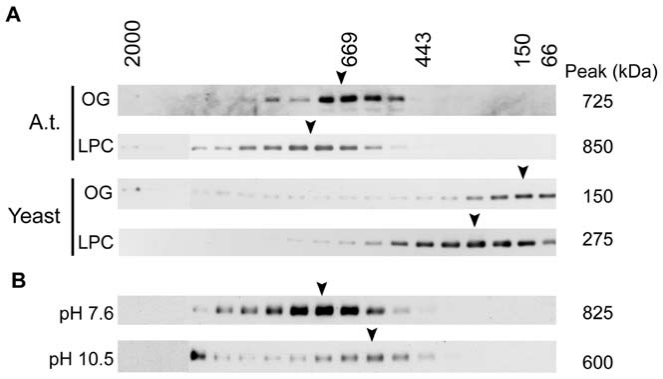
Gel-filtration analysis of ETR1 isolated from its native Arabidopsis or after transgenic expression in yeast. Microsomal fractions were solubilized with octylglucoside (OG) or lysophosphatidyl choline (LPC), and the proteins fractionated on Superose 6HR. ETR1 was detected by immunoblot analysis of the fractions. The estimated molecular mass of the ETR1 complex is indicated to the right of each immunoblot. Positions of the molecular mass markers used to calibrate the column are indicated above. (A) Elution profile of ETR1 from Arabidopsis (A.t.) from plants grown in liquid culture or after transgenic expression in yeast. (B) Effect of pH treatment upon size of the ETR1 complex from Arabidopsis. Microsomes were treated with buffers of either pH 7.6 or 10.5 prior to LPC-solubilization and gel-filtration analysis.

To determine the native size of the ETR1 protein complex, membrane proteins from Arabidopsis were solubilized with either lysophosphatidylcholine (LPC), an ionic phospholipid containing a single fatty acid chain, or octylglucoside (OG), a nonionic detergent [30]. LPC-solubilized ETR1 retains its ability to bind ethylene [11], and thus LPC has the potential ability to preserve the native structure and function of ETR1 and its associated proteins. Solubilized proteins were separated by gel filtration using Fast Protein Liquid Chromatography (FPLC), fractions collected, and the presence of ETR1 determined by immunoblot analysis (Figure 1*A*). ETR1 elutes as part of a protein complex of 725 kDa in the presence of OG and of 850-kDa in the presence of LPC. The difference in size of the complexes is consistent with the larger micelle size of LPC (∼100 kDa) compared to OG (8 kDa) [30]. The size of the high-molecular-mass protein protein complex identified by FPLC is substantially greater than the predicted molecular mass for the disulfide-linked ETR1 homodimer (164 kDa).

Proteins can sometimes fractionate by gel filtration with an apparent molecular mass greater than that predicted from sequence, potentially due to deviations from a globular structure [31]. We took two approaches to rule out this possibility with ETR1. First, we examined the size of the ETR1 complex when transgenically expressed in yeast. Yeast was chosen because ETR1 was previously demonstrated to be functional when transgenically expressed in yeast based on (1) its ability to bind ethylene and (2) its enzymatic His-kinase activity [10], [11], [16], [17]. Yeast should, however, lack proteins found in Arabidopsis that contribute to the formation of an ETR1 protein complex. As shown in Figure 1A, ETR1 solubilized from yeast membranes elutes at 150 kDa in the presence of OG and at 275 kDa in the presence of LPC. In OG, the apparent molecular mass of ETR1 is consistent with the calculated mass of the ETR1 dimer (164 kDa), indicating that ETR1 does not migrate anomalously and that OG contributes very little to the apparent molecular mass of ETR1. The increased apparent molecular mass of ETR1 in the presence of LPC is consistent with the addition of 125 kDa from the detergent micelle to the ETR1 dimer.

As an alternative approach to demonstrate that ETR1 isolated from Arabidopsis is part of a multiprotein complex, we tested the ability of base treatment to remove peripheral proteins from ETR1. For this purpose, Arabidopsis membranes were treated with either sodium carbonate buffer (pH 10.5) or with Tris buffer (pH 7.6) as a control, and the effect of the treatment on LPC-solubilized ETR1 assessed by FPLC (Figure 1B). After treatment with pH 10.5, a significant portion of the ETR1 complex was found to migrate at a molecular mass of 600 kDa, a decrease of 225 kDa compared to the control treatment. The decrease in molecular mass is consistent with the removal of associated proteins from the ETR1 complex by base-treatment. A portion of ETR1 was also observed to migrate at a higher apparent molecular mass upon treatment with base, probably the result of aggregation or denaturation brought on by the harsh treatment.

These data indicate that ETR1 is part of a high-molecular-mass multiprotein complex when isolated from Arabidopsis membranes. Based on FPLC analysis in the presence of OG, the native size of the protein complex is 725 kDa, which is considerably larger than the ETR1 dimer and is therefore consistent with ETR1 being part of a protein complex. Although OG does not contribute significantly to the size of the ETR1 protein complex, OG solubilized only about 10% of total ETR1, compared to greater than 90% solubilized with LPC. Based on its greater efficiency for solubilization and its demonstrated ability to preserve function of ETR1, we used LPC for subsequent experiments.

### The Ethylene Receptors ERS1, ETR2, EIN4, and ERS2 Form Protein Complexes

**Figure 2:**
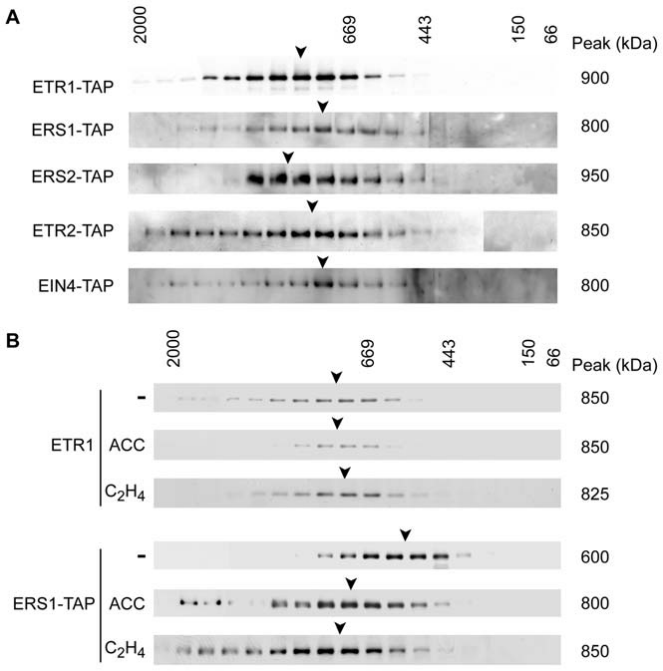
Protein complexes formed by subfamily-1 and subfamily-2 ethylene receptors. (A) Ethylene-receptor protein complexes isolated from plants grown in liquid culture. TAP-tagged versions of the receptors were transgenically expressed in Arabidopsis, solubilized from microsomes by LPC, and analyzed by gel-filtration. TAP-tagged proteins were detected using a rabbit anti-goat IgG antibody coupled to horse-radish peroxidase. (B) Ligand-mediated effects upon the ERS1 and ETR1 ethylene receptor protein complexes. Four-day-old etiolated seedlings from wild-type or ERS1-TAP transgenic plants were treated with the ethylene biosynthesis precursor 50 µM ACC or with 10 µL/L ethylene (C_2_H_4_). The ACC treatment was for four days while the ethylene treatment was for 6 hr. Differences of 50 kDa or less are not significant.

To determine if other members of the ethylene receptor family, besides ETR1, formed protein complexes in Arabidopsis, we generated C-terminal tagged versions of each receptor and expressed these in Arabidopsis. Based on FPLC analysis, the LPC-solubilized receptors were all found to be components of protein complexes, although the size of the protein complex did not directly correlate with the size of the receptor (Figure 2A). For example, both ERS1 and ERS2 are of similar molecular mass, but the size of their protein complexes differed by 150 kDa. The variable size of the protein complexes suggests that there is heterogeneity in their composition, with different receptors potentially able to assemble different multiprotein complexes. Alternatively, different receptors made bind the same associated protein but with different affinities, such that the protein is more readily lost from a complex during solubilization in some cases.

Ligand binding can induce assembly of multiprotein complexes by receptors [32], [33]. We therefore tested the effect of ethylene treatment on the size of the ethylene receptor protein complexes. For this purpose, we performed experiments using dark-grown seedlings because this is a growth condition that displays a pronounced and well-characterized ethylene response [3], [34], [35]. Etiolated seedlings were grown in the absence or presence of aminocyclopropane carboxylic acid (ACC), a precursor of ethylene biosynthesis. Alternatively, to examine a short-term response, seedlings were treated for 6 hr with 10 µL/L ethylene. FPLC analysis showed that the ETR1 protein complex was of similar size to that found in our previous analyses using plants grown in liquid culture and had no significant change in size in response to either the ACC or ethylene treatment (Figure 2B). In contrast, the ERS1 protein complex in etiolated seedlings (Figure 2B) differed in size from what was found when plants were grown in liquid culture (Figure 2A), indicating that growth conditions may affect composition of the ERS1 protein complex. In addition, the ERS1 protein complex also increased by 200–250 kDa in response to growth on ACC or the 6 hr ethylene treatment (Figure 2B), consistent with a ligand-induced change in protein components of the ERS1 protein complex. These data indicate that receptors may form qualitatively different protein complexes, even the closely related subfamily-1 receptors ETR1 and ERS1, which raises the possibility that receptors could participate in unique, non-overlapping regulation of downstream responses.

### Disulfide Bonds Contribute to Maintenance of the ETR1 Protein Complex

**Figure 3:**
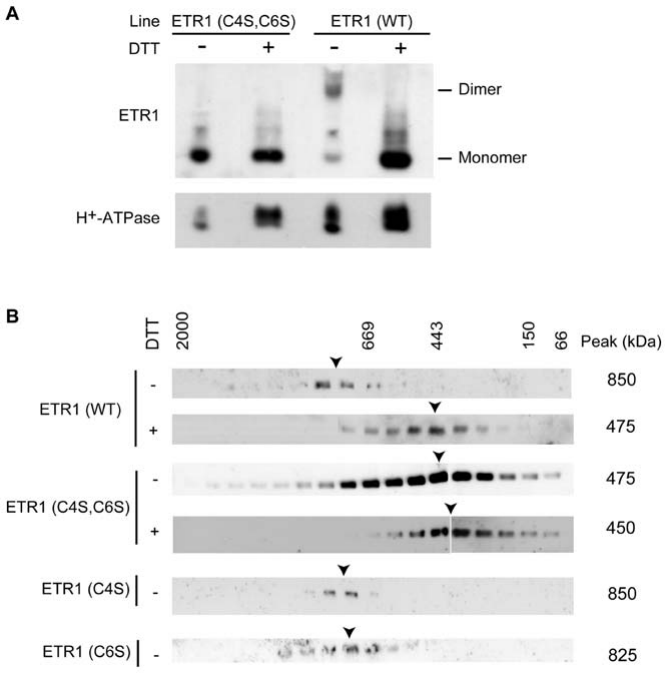
Effect of reduction and cysteine mutations on the size of the ETR1 protein complex. (A) Mutation of Cys4 and Cys6 of ETR1 prevents formation of the disulfide-linked dimer. LPC-solubilized microsomes from plants expressing ETR(wt) or ETR1(C4S,C6S) were incubated in SDS-PAGE loading buffer in the presence or absence of 300 mM DTT, then separated by SDS-PAGE and analyzed by immunoblot. (B) Gel-filtration analysis of wild-type (wt) and cysteine mutants of ETR1. LPC-solubilized microsomes from the indicated plant lines were fractionated on Superose 6HR and the elution profile for ETR1 determined by immunoblot analysis. 5 mM DTT was included in the solubilization and FPLC buffers as indicated.

Previous work has demonstrated that the ETR1 homodimer is linked by disulfide bonds based on its sensitivity to reducing agents such at dithiothreitol (DTT) (Figure 3A) [13]. To determine the role of disulfide bonds in stabilizing the larger ETR1 protein complex from plants, solubilized membrane proteins from Arabidopsis were treated with the reducing agent dithiothreitol (DTT) and examined by FPLC. In the presence of DTT, the ETR1 receptor complex displayed a molecular mass of 475 kDa (Figure 3B), approximately half the size of the non-reduced complex, suggesting that disulfide bonds are important for maintaining the stability of the ETR1 receptor complex.

To determine if the DTT treatment was reducing the disulfide bonds linking together the ETR1 homodimer or removing additional proteins associated with ETR1 through disulfide bonds, we employed mutant versions of ETR1. Based on evidence obtained by transgenic expression of ETR1 in yeast, Cys4 and Cys6 are important for formation of the disulfide-linked ETR1 homodimer [13]. We therefore used site-directed mutagenesis of ETR1 to change Cys4 and Cys6 to Ser, producing ETR1(C4S), ETR1(C6S), and ETR1(C4S,C6S), and expressed these mutant versions of ETR1 in the *etr1-6 etr2-3 ein4-4* background. All three mutant versions of ETR1 rescued the constitutive-ethylene response phenotype of *etr1-6 etr2-3 ein4-4*, indicating that they are still functional receptors, consistent with previously published results [36]. We also confirmed that ETR1(C4S,C6S) isolated from plants was unable to maintain a disulfide-linked homodimer by examining the receptor size with reducing and non-reducing SDS-PAGE (Figure 3A).

Solubilized ETR1(C4S,C6S) was subjected to FPLC analysis and the apparent molecular mass of the complex was determined to be 475 kDa, which is approximately half the molecular mass of the wild-type ETR1 receptor complex (Figure 3B). In the presence of 5 mM DTT the ETR1(C4S, C6S) complex did not reduce further as shown by essentially the same elution profile of ETR1(C4S, C6S) in the absence of DTT (Figure 3B). In contrast to the results obtained with ETR1(C4S,C6S), the ETR1(C4S) and ETR1(C6S) mutants behaved similarly to wild-type ETR1 when the complexes were examined by gel filtration, demonstrating roles for both Cys4 and Cys6 in maintaining the high-molecular-mass ETR1 protein complex. Taken together, these data demonstrate that DTT treatment reduces two disulfide bonds necessary for covalently linking the ETR1 homodimer, and that these bonds are important for maintenance of the homodimer during solubilization, with a consequence that the bonds also serve to stabilize the high-molecular-mass protein complex during solubilization.

### Role of Disulfide Bonds in Stabilizing Ethylene-Binding Capacity of ETR1

**Figure 4:**
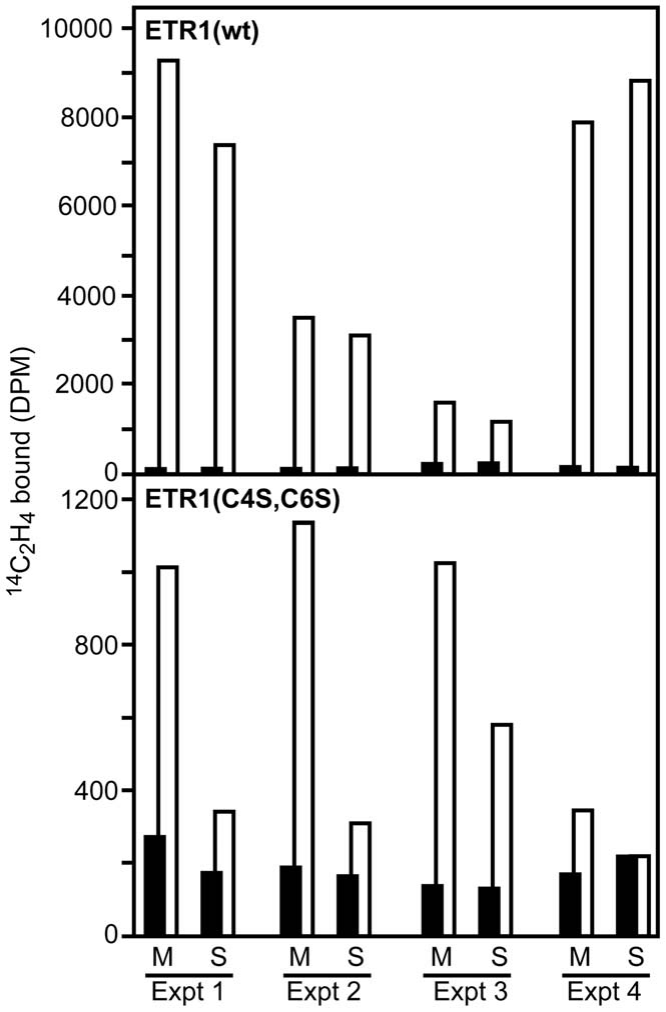
Effect of ETR1 disulfide bonds on the ability of the receptor to bind ethylene. Membranes were isolated from transgenic yeast expressing either wild-type ETR1 (wt) or ETR1(C4S,C6S). Each membrane sample was separated into equal portions, and portions incubated in the absence (M) or presence of 5 mg/mL SB-16 to solubilize the receptor (S), prior to being examined for ethylene binding. Saturable ethylene binding is indicated as the difference between samples treated with ^14^C-ethylene (white bars) and identical samples treated with ^14^C-ethylene and excess ^12^C-ethylene (overlapping black bars). Results from four independent experiments are shown, with duplicate samples being examined in each experiment.

Our data indicate that solubilization of ETR1(C4S,C6S) disrupts the ETR1 homodimer. There is one ethylene binding site per homodimer, the current model support a binding site containing a single requisite copper ion liganded by both polypetides of the homodimer [4], [10], [11], [12]. Thus solubilization of the ETR1(C4S,C6S) mutant and consequent loss of the homodimeric form is predicted to disrupt ethylene binding. We directly tested this prediction by using wild-type ETR1 and ETR1(C4S,C6S) expressed in a transgenic yeast system (Fig. 4), previously shown to allow for expression of functional ethylene receptors [10], [11], [16], [22]. Membranes were isolated and each membrane sample separated into equal portions, one portion being solubilized by the addition of detergent and the other portion being left intact. Solubilization had mimimal effect upon ethylene binding by wild-type ETR1. In contrast, solubilization resulted in a marked decrease in the ability of ETR1(C4S,C6S) to bind ethylene. The residual binding ability found in the mutant is likely due to the persistence of a small amount of dimeric ETR1(C4S,C6S) maintained through non-covalent interactions. These data support a role for the disulfide bonds in stabilizing the ETR1 homodimer and indicate that their disruption can result in a receptor with reduced ethylene-binding capacity.

### Multiple Domains of ETR1 Are Required for Formation of the Protein Complex

**Figure 5:**
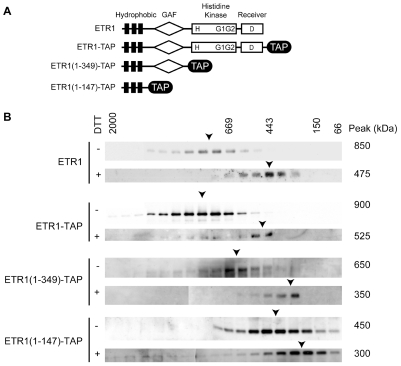
Effect of truncations in ETR1 on the size of the ETR1 protein complex. (A) Features of ETR1 constructs. Positions of transmembrane domains (black rectangles), GAF domain (diamond), His-kinase and receiver domains (rectangles), and the TAP tag (black oval) are indicated. (B) Gel filtration profiles of full-length and truncated versions of ETR1. Microsomes were solubilized with LPC and fractionated, 5 mM DTT being added as indicated. TAP-tagged versions of ETR1 were detected using a rabbit anti-goat IgG antibody coupled to horse-radish peroxidase.

ETR1 is a modular protein, composed of a hydrophobic domain near the N-terminus, a GAF domain, a His kinase (HK) domain, and a receiver (R) domain [3], [37], [38]. To determine which regions of ETR1 are involved in formation of the protein complex and to provide further evidence for the location of the disulfide bonds necessary for ETR1 dimer linkage, truncated versions of ETR1 were constructed, transgenically expressed in Arabidopsis, and the size of the resulting protein complex determined by FPLC analysis (Figure 5). To avoid complications due to native full-length ETR1, the *etr1-7* null mutant was used as the genetic background [39], [40].

We used C-terminal tags to allow for immunological detection of truncated forms of ETR1. For this purpose, versions of the Tandem Affinity Purification (TAP) tag [41] were added to the C-terminal end of full-length ETR1 and the two truncated ETR1 constructs ETR1(1-147) and ETR1(1-349) (Figure 5A). ETR1(1-147)-TAP contains only the N-terminal transmembrane domains of ETR1, but still contains a functional ethylene binding site based on the ability of an ETR1(1-128)-GST fusion to bind ethylene when transgenically expressed in yeast [11]. ETR1(1-349)-TAP is a truncated version of ETR1 containing transmembrane and GAF domains, but lacking the His kinase and receiver domains [42]. ETR1-TAP fractionated by FPLC at approximately 50 kDa larger than the non-tagged versions (Figure 5B). This slight increase in molecular mass can be accounted for by the two TAP tags that would be found in the ETR1 dimer and indicates that the TAP tag does not interfere with the ability of ETR1 to form a protein complex. The gel-filtration data for the truncated versions of ETR1 support a domain-based organization to the protein complex (Figure 5B). ETR1(1-147)-TAP formed a complex of 400 kDa. From this number was subtracted the contribution of LPC/lipid (125 kDa) and the ETR1(1-147)-TAP dimer (90 kDa), leaving approximately 200 kDa due to unaccounted components of the complex associated with the transmembrane domains. Similarly, we calculate that approximately 200 kDa additional proteins are associated with the GAF domain and about 150 kDa with the His kinase/receiver domains. These data suggest that the ETR1 receptor complex is assembled in a domain-specific manner, with each domain required for the assembly of independent components of the receptor complex.

We also examined the effects of reducing agents upon the complexes formed by the different versions of ETR1, this serving as an independent means to determine location of the disulfide bonds that maintain the ETR1 dimer (Figure 5B). Solubilization of the ETR1-TAP receptor in the presence of 5 mM DTT results in a reduction in mass from 900 kDa to approximately 525 kDa as determined by FPLC analysis, demonstrating that the TAP tag does not interfere with the ability of DTT to reduce the complex size. When the ETR1(1-349)-TAP receptor complex was treated with DTT, the complex was reduced from 650 kDa to approximately 350 kDa. This reduction to approximately half of the non-reduced receptor suggests that the DTT treatment is cleaving the disulfide bonds necessary for ETR1 dimer linkage. A similar result was observed for DTT treatment of the ETR1(1-147)-TAP receptor. The non-reduced form was determined to have a molecular mass of approximately 450 kDa, and DTT treatment reduced the molecular mass to 300 kDa (Figure 5B). Note that it is necessary to subtract the mass of the micelle (125 kDa) to calculate the difference in mass between the non-reduced and reduced protein complexes. After performing this calculation it is apparent that the molecular mass of 175 kDa for the reduced ETR1 (1-147)TAP receptor is almost half of the molecular mass of 325 kDa for the non-reduced receptor. This deletion analysis of the ETR1 receptor confirms that the disulfide bonds necessary for maintaining the ETR1 complex are located within the N-terminal domain of ETR1, consistent with the proposed role for Cys4 and Cys6.

### Requirements for ER-Localization Are within the N-Terminal Region of ETR1

**Figure 6:**
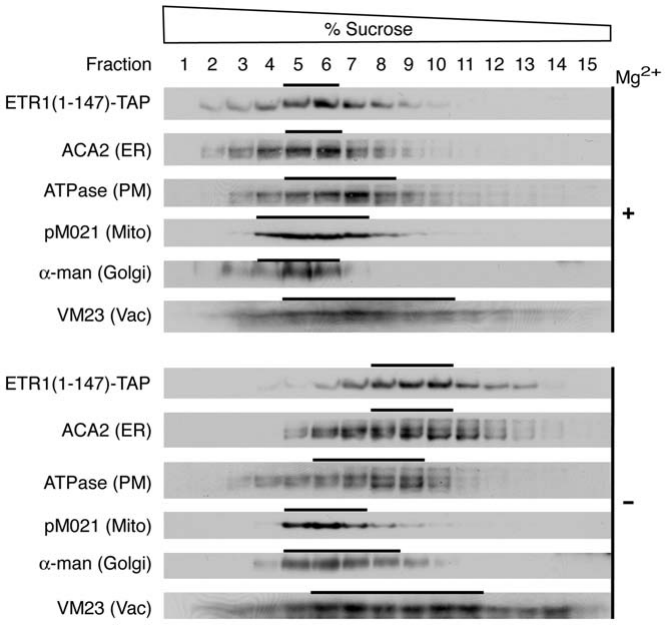
Localization of ETR1(1-147)-TAP to the endoplasmic reticulum based on analysis by sucrose density gradient centrifugation. Arabidopsis membranes were fractionated over 20–50% (w/w) sucrose gradients. Gradients were run in the presence of Mg (+) to stabilize membrane-associated proteins or in the absence of Mg (−) to dissociate membrane-associated proteins. Samples (20 µL) of each fraction were analyzed by immunoblot for ETR1(1-147)-TAP, the ER marker ACA2, the PM marker H^+^-ATPase, the mitochondrial inner membrane marker F1-ATPase (pM021), the Golgi marker α-mannosidase, and the vacuole marker VM23.

A potential concern with the analysis of the truncated versions of ETR1 is whether they are localized in the correct intracellular context for formation of the protein complex. To determine if the truncated ETR1(1-147)-TAP still localized to the ER like full-length ETR1 [7], the subcellular membrane localization of ETR1(1-147)-TAP was determined by sucrose density gradient centrifugation (Figure 6). Centrifugation was performed in the presence and absence of Mg^2+^ to allow for the discrimination of ER-associated proteins. Association of ribosomes with the ER is Mg^2+^-dependent, so removal of Mg^2+^ results in dissociation of ribosomes from the ER and a diagnostic redistribution of ER from higher to lower density on the gradient [43]. Fractions from the sucrose gradient were analyzed by immunoblot for the presence of ETR1(1-147)-TAP as well as for markers specific for PM, mitochondria, tonoplast, Golgi, and ER (Figure 6). The majority of ETR1(1-147)-TAP exhibited a strong Mg^2+^-dependent density-shift from 43–46% to 34–39% (w/w) sucrose, similar to that observed for the ER marker ACA2. The distribution of ETR1(1-147)-TAP could be differentiated from the plasma membrane marker (H^+^-ATPase), the mitochondrial inner membrane marker (pM021), the tonoplast marker (VM23), and the Golgi marker (α-mannosidase), which did not demonstrate the same Mg^2+^-induced shift. ETR1(1-147)-TAP also did not correlate with the chloroplast thylakoid marker (chlorophyll absorbance), which peaked at 46% and 45% in the presence and absence of Mg^2+^, respectively (results not shown). These data indicate that ETR1(1-147)-TAP localizes to the appropriate location for formation of the ETR1 protein complex, and also indicate that the determinants for ER localization and retention are found within the first 147 amino acids of ETR1.

### Effect of Ethylene Pathway Mutations upon Formation of the ETR1 Protein Complex

**Figure 7:**
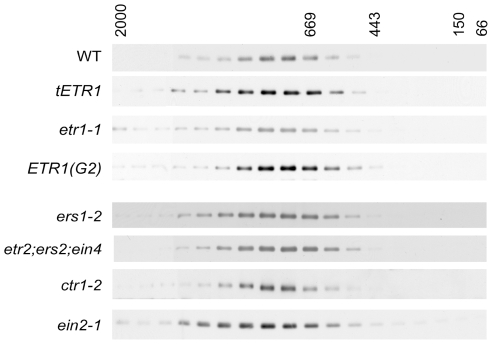
Effect of ethylene pathway mutations on the size of the ETR1 protein complex. LPC-solubilized microsomes were fractionated on Superose 6HR and the elution profile for ETR1 determined by immunoblot analysis. Analysis was performed in a line where additional copies of ETR1 was trangenically expressed (tETR1), in a line containing an ethylene-insensitive mutation in ETR1 (*etr1-1*), and in a line containing a kinase-deficient version of ETR1 (ETR1-G2). Analysis was also performed in lines containing mutations in other members of the ethylene receptor family (the single mutant *ers1-2* and the triple mutant *etr2/ers2/ein4*), and in the downstream pathway components *CTR1* and *EIN2*.

To gain further information on the requirements for formation of the protein complex, we examined the effects of additional perturbations in ETR1 expression and function (Figure 7). To examine the effect of increased ETR1 expression level, we used a transgenic line (tETR1) transformed with an additional genomic copy of the *ETR1* gene, which results in a 4-fold increase in the level of immunodetectable ETR1 [7]; no change in the size of the ETR1 protein complex were observed in this line indicating that other components of the protein complex were not limiting at this increased expression level. We tested two mutants of ETR1 for their effect upon formation of the protein complex. In the mutant *etr1-1*, ethylene binding by the receptor is abolished due to a missense mutation (Cys65Tyr) in the ethylene-binding site [10], [11]. In the mutant *ETR1(G2)*, His-kinase activity is lost due to a mutation within the ATP binding site of the receptor [42]; this mutation has a modest effect upon the plant's ethylene response indicating that kinase activity is likely to modulate rather than be essential for signaling [21]. Both the etr1-1 and the ETR1(G2) protein complexes were similar in size to that of wild-type ETR1, indicating that neither a functional ethylene binding site nor a functional kinase domain is required for assembly of the protein complex.

Loss of an obligate component of a protein complex should result in a decrease in the size of the protein complex. We therefore determined the size of the ETR1 protein complex in mutant backgrounds containing loss-of-function mutations in known components of the ethylene signal transduction pathway (Figure 7). These included a triple mutant line for the ethylene receptors of subfamily 2 (*etr2*/*ers2*/*ein4*) [39], the other ethylene receptor of subfamily 1 (*ers1-2*) [18], [21], the Raf-like kinase CTR1 (*ctr1-2*) [44], and the Nramp-like protein EIN2 (*ein2-1*) [45]. The *ctr1-2* mutation is a frameshift that lacks detectable protein based on immunoblot analysis [27]; the *ein2-1* mutation results in a premature stop codon predicted to eliminate 80 kDa from the encoded protein [45]. We also examined another null mutation (*ctr1-9*) and a missense mutation predicted to reduce kinase activity (*ctr1-4*) in CTR1 (results not shown) [26], [44]. In no case did we observe a significant reduction in size of the ETR1 protein complex as determined by FPLC analysis, indicating that none of these proteins forms an obligate component of the complex we have identified by gel-filtration analysis.

## Discussion

Signal transduction involves protein-protein interactions and thus receptors typically function as multicomponent complexes or protein complexes [32], [33]. We find that all five ethylene receptors of Arabidopsis are solubilized from membranes as high-molecular-mass protein complexes, consistent with a protein complex being the functional unit for ethylene perception and signal transduction. Among the types of protein-protein interactions possible in a complex are homo- and hetero-oligomeric interactions, non-obligate and obligate interactions, and transient and permanent interactions [46]. Characterization of the ethylene receptors indicates that multiple types of interactions play roles in formation of the protein complex.

Our data support a model in which the solubilized ETR1 receptor protein complex contains a receptor homodimer as the predominant receptor form. We consider a dimer rather than a monomer as the minimal receptor component of the complex because a disulfide-linked homodimer is the functional unit for ethylene perception [11], [13] and because treatment with reducing agents halves the size of the complex. Under all conditions examined, the ethylene receptor protein complexes were significantly larger than that predicted for the homodimer, indicative that the receptors form additional stable protein-protein associations. Deletion analysis indicates that ETR1 has multiple binding sites for components of the complex, thereby supporting a modular organization to the protein complex.

Previous work has demonstrated that ethylene receptors are capable of forming higher order interactions with other members of the receptor family [9], [29] as well as with the Raf-like kinase CTR1 [24], [25], [26], [27]. In addition, initial analysis also suggests that the receptors interact with EIN2, although this result needs confirmation at native levels of expression [28]. However, these interactors do not appear to significantly contribute as obligate components of the complexes identified by FPLC in this study. This conclusion is based on the following observations. First, the size of the ETR1 complex is not affected by null mutations in the other receptors, CTR1, or EIN2. Second, when ETR1 is transgenically expressed in yeast, the solubilized receptor is a dimer based on gel filtration analysis, indicating that ETR1 dimers do not form stable higher order interactions under these solubilization conditions. Third, we find that the ETR1 and ERS1 protein complexes are of different sizes and have a qualitatively different response to ethylene, indicating substantial independence between the ETR1 and ERS1 protein complexes. It is likely that these higher order interactions are not stably preserved during solubilization, a possibility consistent with prior work in which we saw that, although we could pull down CTR1 and other ethylene receptors with ETR1 following solubilization [27], [29], longer term incubation in the presence of detergent resulted in decreased recovery of the interactors. It is also possible that a portion of the solubilized receptors are present as higher order complexes but these represent a small percentage of the total, or that the higher order complexes are so large that they are not resolved by the FPLC analysis. Thus, the proteins we find associated with the solubilized ethylene receptor complexes are likely to represent novel components not previously identified based on genetic analysis.

Whereas some components may form stable associations with a receptor, others are reversibly nucleated upon ligand binding [32], [47]. We find that the ERS1 protein complex, in contrast to the ETR1 protein complex, dynamically changes in response to the ligand ethylene, consistent with the binding of additional transiently-associated protein components. The effect of ethylene upon the ERS1 complex is reminiscent of ligand-induced changes in complexes formed by animal receptor tyrosine kinases, where ligand binding induces autophosphorylation and the recruitment of proteins that bind to the phosphorylated sites [32], [47]. Phosphorylation could potentially play a similar role in regulating transient participation of proteins in the ERS1 protein complex, whether it is autophosphorylation mediated by ERS1 or intermolecular serine/threonine phosphorylation mediated by the associated CTR1 protein kinase.

Based on the FPLC analysis, an open question remains as to what additional elements associate with the receptors. The native expression level of the ethylene receptors is very low, which has to date rendered purification to homogeneity from plants impractical for the identification of interacting components. We have had some success in overexpressing and purifying portions of the receptors from plants, but these did not yield significant levels of associated proteins (Gao and Schaller, unpublished data), suggesting that contributors to the complexes may be expressed at similarly low levels as the receptors. It is thus likely that for the near future the greatest progress in identification of elements of the receptor complexes will be made by first identifying potential interactors through genetic or two-hybrid type screens and then confirming these interactions *in planta*. Along these lines it is possible that RTE1, which has recently been found to regulate ETR1 activity and localize to the same subcellular membrane system, represents one such element [48], [49]. However, it should be noted that due to its small size (28 kDa) and the modular nature of interactions with the receptor, RTE1 is unlikely to substantially contribute to the size of the receptor complex and that additional elements remain to be discovered.

Our data provide new information on the role of disulfide bonds in stabilizing the ETR1 structure. Previous work has demonstrated ethylene receptors form disulfide-linked homodimers [10], [14], [50], with work in transgenic yeast supporting a role for Cys4 and Cys6 of ETR1 in maintaining these covalent linkages [10]. Our data confirm that ETR1 exists as covalent homodimer *in planta* and demonstrate that both Cys4 and Cys6 are involved in making disulfide bonds in the native protein. Although the ethylene receptors exist as covalently-linked homodimers in plants the role of this covalent linkage in ethylene signaling has been unclear. Mutant versions of ETR1 in which the cysteines were mutated to alanine rescued the constitutive ethylene-response phenotype found in the *etr1-7; ers1-2* double mutant, indicating that non-covalent interactions are sufficient to form and maintain active receptor dimers [36]. We found that the disulfide bonds were required for maintenance of the receptor homodimers and functionality under solubilization conditions. These data support a role for the disulfide bonds in folding and stability of the receptors, consistent with their typical role in other proteins [51]. The disulfide bonds may facilitate assembly of the homodimer during translation, particularly given their presence at the N-terminus, in which case they could potentially increase the rate of formation and/or the percentage of functional receptors. They could potentially also stabilize the protein under conditions of stress and, as such, their role may not be obvious under optimal growth conditions.

The truncation analysis of ETR1 indicates that the sequences required for both targeting to the secretory system and retention at the ER are contained within the N-terminal 147 amino acids of ETR1. This region of ETR1 encompasses the three transmembrane segments of ETR1. ETR1 does not contain a predicted signal sequence and thus information for targeting to the secretory system is likely to be contained within its first transmembrane segment [52], which would in this case function as an uncleaved signal sequence. Retention of ETR1 at the ER could potentially be mediated by interactions of the transmembrane domain with other proteins, such as those revealed by the truncation analysis. Alternatively, the length of the transmembrane segments themselves may influence the final destination of transmembrane proteins within the secretory pathway [53].

Our results support a substantial degree of heterogeneity among the ethylene receptor protein complexes. Different members of the ethylene receptor family (e.g. ETR1 and ERS1) form protein complexes of different sizes indicating that, under the same conditions for plant growth and subsequent protein isolation, that the receptors exhibit a preferential association with some different binding partners. It is not clear at this point how much heterogeneity there is in the makeup of individual receptor complexes. It is reasonable, however, that ETR1 could participate in protein complexes with different binding partners. The heterogeneity uncovered through the analyses reported here may allow for the tailoring of ethylene receptor protein complexes to particular cellular tasks.

## Materials and Methods

### Constructs and Transformation

For preparation of full length ETR1 with a C-terminal tandem-affinity protein (TAP)^1^ tag, a binary vector (pCAMBIA1380-TAP) was prepared for expression of affinity-tagged proteins in *Arabidopsis*. The TAP tag was amplified from the vector pBS1479 [41] and cloned into the *BamH* I and *Hind* III restriction sites of the vector pCAMBIA1380 (GenBank accession no. AF234301). The region encoding ETR1 along with upstream promoter sequence was amplified from a 7.3-Kb genomic clone [37] using 5′-primer GGATCCAGTGGTTCCAACTCGGGA and 3′-primer GGATCCCATGCCCTCGTACAGTAC. The PCR product was cloned into the *BamH*I site of pCAMBIA1380-TAP to make pCAMBIA-ETR1-TAP. For preparation of the truncated versions of ETR1 with the C-terminal TAP tag, the regions encoding ETR1(1-349) and ETR1(1-147) along with upstream promoter sequence were amplified from pCAMBIA-ETR1-TAP using 5′-primer GTCGACAGTGGTTCCAACTCGGGA and 3′-primer GTCGACCCGCTAGGAAATCATTGC for ETR1(1-349) and -3′primer GTCGACTTCTCACATGCCTTCCGG for ETR1(1-147). The PCR products were then cloned into the *Sal*I site of pCAMBIA2380-myc-TAP [27] to yield constructs with the c-Myc epitope in tandem with the original TAP tag. Site-directed mutagenesis of Cys4 and Cys6 to generate the ETR1(C4S, C6S), ETR1(C4S) and ETR1(C6S) mutants was performed as previously described [18], [54].

For transformation into Arabidopsis, constructs were introduced into *Agrobacterium tumefacians* strain GV3101 and used to transform Arabidopsis by the floral-dip method [55]. The tagged versions of ETR1 were transformed into the *etr1-7* loss-of-function mutant background [39]. The ETR1(C4S, C6S), ETR1(C4S) and ETR1(C6S) mutants were transformed into an *etr1-6 etr2-3 ein4-4* Arabidopsis triple mutant background, their ability to rescue the mutant phenotype indicating that they are functional receptors.

Construction and transformation of Arabidopsis with C-terminal TAP-tagged versions of ERS1, ETR2, ERS2, and EIN4 was previously described [29]. Construction and transformation of Arabidopsis with the ETR1 constructs tETR1, ETR1(1-349), and ETR1(G2) was also previously described [12], [42]. Preparation of transgenic yeast expressing full-length and mutant versions of ETR1 was as described [13].

### Membrane Fractionation

Microsomal and soluble fractions were isolated from either dark-grown Arabidopsis seedlings [40] or Arabidopsis plants grown in liquid culture under constant light [7]. Aminovinylglycine (AVG), an inhibitor of ethylene biosynthesis, was included in growth media for dark-grown seedlings. Plant material was homogenized in a buffer containing 30 mM Tris (pH 7.6 at 22°C), 150 mM NaCl, 0.1 mM EDTA, and 20% (v/v) glycerol with protease inhibitors and then centrifuged at 5,000×g for 5 min as described [7], [40]. The supernatant was then centrifuged at 100,000×g for 30 min, and the resulting membrane pellet resuspended in 10 mM Tris (pH 7.6 at 22°C), 150 mM NaCl, 0.1 mM EDTA, and 10% (v/v) glycerol with protease inhibitors (resuspension buffer).

Base treatment of Arabidopsis membranes was performed according to Millar and Heazlewood [56]. The membrane pellet was resuspended at 1.5 mg/mL protein in resuspension buffer buffered with either 100 mM Tris (pH 7.6) as the control or with 100 mM Na_2_CO_3_ (pH 10.5) for the base treatment. Samples were incubated for 30 min at 4°C, then centrifuged for 30 min at 100,000×g, and the membrane pellet was resuspended in resuspension buffer (pH 7.6) for solubilization.

Sucrose density gradient centrifugation of Arabidopsis membranes was performed as described [7] using 20-50% (w/w) sucrose gradients in 10 mM Tris (pH 7.6), 1 mM DTT, 2 mM EDTA, and 0.1 mM PMSF. For analyses performed in the presence of Mg^2+^, 5 mM MgCl_2_ was added to homogenization, resuspension, and centrifugation buffers. Gradient fractions were analyzed for the presence of the ER, PM, mitochondrial inner membrane, tonoplast, and Golgi by immunoblot using antibodies that recognized specific membrane markers. Thylakoid membranes were identified by spectrophotometric analysis of chlorophyll levels [57].

### FPLC Analysis of Solubilized Membrane Proteins

To solubilize Arabidopsis membrane proteins, the membrane resuspension buffer was supplemented with 0.5% (w/v) 1-Palmitoyl-2-hydroy-*sn*-Glycero-3-Phosphocholine (LPC) (Avanti Polar Lipids, Inc) or 0.6% (w/v) Octyl β-D-glucopyranoside (OG) (Sigma). For reduction of membrane proteins, 5 mM dithiothreitol (DTT) was included in the solubilization buffer. The protein concentration was adjusted to 1.5 mg/ml, incubated for 1 hr at 4°C, and then centrifuged at 100,000×g for 30 min. The supernatant was passed through a 0.2-µm filter then immediately injected into a fast protein liquid chromatography (FPLC) system (Amersham Biosciences) equipped with a Superose 6 10/30 column (Pharmacia). The column was eluted with the resuspension buffer containing 10-fold reduced detergent concentrations, and 0.5 ml fractions collected. The column was calibrated with the gel filtration molecular weight markers carbonic anhydrase (29 kDa), bovine serum albumin (BSA, 66 kDa), alcohol dehydrogenase (150 kDa), β-amylase (200 kDa), apoferritin (443 kDa), thyroglobulin (667 kDa), and blue dextran (2000 kDa) (Sigma).

Total yeast protein was isolated from cells transgenically expressing ETR1 [13] by beating with glass beads in homogenization buffer as described [16]. After centrifugation for 5 min at 3,000×g, the supernatant was brought to 0.5% (w/v) LPC or 0.6% (w/v) OG. The protein concentration was adjusted to 1.5 mg/ml, incubated for 1 hr at 4°C, and then centrifuged for 30 min at 100,000×g. The supernatant was analyzed by gel filtration using the FPLC system as described above.

### Antibodies and Immunoblot Analysis

Immunoblot analysis was performed as described [42]. Protein concentration was determined by use of the BCA reagent (Pierce) according to the manufacturer after first adding 0.2 mL 0.4% (w/v) deoxycholate to solubilize membrane proteins. BSA was used as a standard for protein assays. Prior to SDS-PAGE [58], protein samples were mixed with SDS-PAGE loading buffer and incubated at 37°C for 1 hr or ramped from 37°C to 73°C over 40 min using a thermocyler, so as to prevent the aggregation of integral membrane proteins that can occur with boiling [13], [57]. Following SDS-PAGE, proteins were electrotransferred to Immobilon nylon membrane (Millipore) for immunoblotting. Immunodecorated proteins were visualized by enhanced chemiluminescence detection according to the manufacturer (Pierce Chemical).

Native ETR1 protein was detected using an antibody generated against amino acids 401-738 of ETR1 [13]. TAP-tagged receptors were detected based on the ability of the protein-A motif to bind rabbit anti-goat IgG antibody coupled to horse-radish peroxidase. Specific Arabidopsis membranes were identified by antibodies against the ER-marker ACA2 [59], the PM-marker H^+^-ATPase [60], the mitochondrial inner-membrane marker F1-ATPase [61], and the tonoplast-marker VM23 [62], and the Golgi marker α-mannosidase I [63] (antibody provided by Sebastian Bednarek, Univ. of Wisconsin-Madison).

### Ethylene Binding Analysis

Membranes were isolated from transgenic yeast as previously described [13], [22], and receptors solubilized when appropriate by incubation of the membranes with 5 mg/mL SB-16 [22]. Saturable ethylene binding was determined as described by incubation of samples in sealed glass chambers containing either ^14^C-ethylene (0.1 µL/L) or ^14^C-ethylene (0.1 µL/L) plus ^12^C-ethylene (100 µL/L) [10], [22].
